# Three-dimensional observation of Virchow–Robin spaces in the basal ganglia and white matter and their relevance to idiopathic normal pressure hydrocephalus

**DOI:** 10.1186/s12987-015-0010-1

**Published:** 2015-06-26

**Authors:** Masatsune Ishikawa, Shigeki Yamada, Kazuo Yamamoto

**Affiliations:** Department of Neurosurgery, Rakuwakai Otowa Hospital, 2 Chinji-cho, Otowa, Yamashina-ku, Kyoto, 607-8062 Japan; Department of Normal Pressure Hydrocephalus Center, Rakuwakai Otowa Hospital, 2 Chinji-cho, Otowa, Yamashina-ku, Kyoto, 607-8062 Japan

**Keywords:** Virchow–Robin spaces, Perivascular spaces, Normal pressure hydrocephalus, Interstitial fluid, Magnetic resonance imaging

## Abstract

**Background:**

Virchow–Robin spaces (VRS) are brain perivascular spaces containing perforating arteries. Although enlarged VRS are associated with various disorders such as Alzheimer’s disease, cerebrovascular disease, and head trauma, their functional role remains unclear. Using highly fluid-sensitive magnetic resonance imaging (MRI) sequences, fine morphological features of VRS and their relevance to idiopathic normal pressure hydrocephalus (iNPH) were investigated.

**Methods:**

Three-dimensional constructive interference in steady state (3D-CISS) on 3 Tesla MRI was applied to 29 individuals. The morphology and number of VRS in the basal ganglia and white matter were compared between 20 patients with iNPH and nine age-matched controls. The VRS number per hemisphere was classified into three grades: few, moderate, and abundant.

**Results:**

Virchow–Robin spaces in the basal ganglia were curved, irregularly sized and shaped, and communicated with the cerebrospinal fluid in the subarachnoid space; they contained perforating arteries. VRS in the white matter were straight, smooth, homogeneously sized and shaped, and did not penetrate the cortex. Arteries were not seen in VRS of the white matter. White matter VRS were sparse in patients with iNPH. In contrast, basal ganglia VRS positively correlated with age. Postoperatively after shunt surgery, VRS in the white matter were mildly decreased in diameter, but not in number. No significant changes were noted in basal ganglia VRS.

**Conclusions:**

The present study revealed different morphological features of VRS in the basal ganglia and white matter. VRS in the basal ganglia were seen as genuine perivascular spaces; while neither communication with subarachnoid spaces nor arteries were seen in white matter VRS, even by 3D-CISS sequences and high-resolution magnetic resonance angiography on 3T-MRI. White matter VRS were sparse in patients with iNPH and they were mildly decreased in diameter, but did not change in number after surgery. At present, it remains unclear whether the white matter VRS are dilated interstitial fluid spaces or cerebral amyloid angiopathy, or both. Further studies are necessary to elucidate the functional role of VRS in normal subjects and patients with iNPH.

## Background

Virchow–Robin spaces (VRS) are regarded as perivascular spaces containing perforating arteries extending into the brain parenchyma [1]. VRS appear as round or tubular shaped high-signal areas on T2-weighted magnetic resonance images (MRI) [2, 3]. They may be as large as 5 mm in diameter in healthy and aged individuals [4] and the diameter increases with age in the basal ganglia and white matter [5]. Various disorders such as Alzheimer’s disease [6], cerebrovascular disease [3, 7, 8] and head trauma [9] are associated with a higher frequency of VRS, but the relevance of VRS frequency to idiopathic normal pressure hydrocephalus (iNPH) is unclear.

Idiopathic NPH is a syndrome of gait disturbance, dementia, and urinary incontinence with ventriculomegaly, which is prevalent among aging individuals. The symptoms of iNPH can be improved by cerebrospinal fluid (CSF) shunt surgery [10, 11] and it develops insidiously with no known causes. It is an important social issue in countries with a large aging population and the pathogenesis of iNPH remains unclear. Recently, interactions between the CSF and interstitial fluid (ISF) have become a major focus in CSF research [12, 13]. Since iNPH is regarded as a disorder of brain fluid regulation, studying whether VRS are relevant to the pathophysiology of iNPH is warranted.

Three-dimensional constructive interference in steady state (3D-CISS) is a gradient-echo MRI sequence that is used to investigate fine morphological features in water-filled spaces with high signal and extremely high spatial resolution [14, 15]. In this study, we examined fine morphological features of VRS in iNPH and control subjects using 3D-CISS sequences on 3-T MRI.

## Methods

### Patients

This study was approved by the Institutional Review Board of Rakuwakai Otowa Hospital, Japan. Written informed consent was obtained from all subjects. Twenty-nine subjects were divided into a control and iNPH group. The control group consisted of nine age-matched individuals who underwent MRI for mild headache of acute or subacute onset. All were independently active and three had been diagnosed with unruptured aneurysms at other hospitals. Otherwise, they maintained normal social activity. The iNPH group comprised 20 patients with probable iNPH. Patients with probable iNPH were defined as having one or more NPH symptoms (gait disturbance, dementia, and urinary incontinence), ventriculomegaly, and a positive response to removal of 30 ml CSF (tap test) [16]. A positive response to the tap test was defined as ≥1-point symptom improvement on the idiopathic normal pressure hydrocephalus grading scale (iNPHGS), ≥10% improvement on the timed up-and-go test, or ≥3-point improvement on the mini-mental state examination with reference to the Japanese iNPH guidelines [17]. The observation of disproportionately enlarged subarachnoid-space hydrocephalus (DESH) [11] on MRI, which consists of ventriculomegaly, high convexity tightness, and an enlarged Sylvian fissure, is useful for selection of patients with probable iNPH. In this study, patients with two of the above three MRI criteria were included if they also showed a positive response to the tap test.

Daily life activity was assessed using the modified Rankin scale (mRS), a widely used scale for patient disability [18]. Gait, cognition, and urinary control were assessed using iNPHGS [19]. The iNPHGS consists of three symptom domains and is divided into Grade 0–4 for each symptom domain: Grade 0—normal, Grade 1—subjective symptoms, Grade 2—mild disturbance, Grade 3—moderate disturbance, and Grade 4—severe disturbance. CSF shunt surgery was performed on 12 patients, resulting in symptom improvement. Eight patients did not undergo surgery because of refusal by the patient or family, high-grade dementia, or severe systemic disorders, such as severe chronic kidney disease or chronic obstructive pulmonary disease.

### MRI

All MRI examinations were performed using a 64-channel 3 Tesla MRI system (MAGNETOM Skyra, Siemens AG, Muenchen, Germany). 3D-CISS sequence analysis and a 3D-T2 weighted SPACE sequence were applied to all MRIs. The latter sequence evaluated CSF volume in the ventricles/subarachnoid spaces and will be reported separately. The parameters of 3D-CISS were as follows: repetition time (TR): 6.26 ms, echo time (TE): 2.70 ms, flip angle: 40°, slice thickness: 0.75 mm, slice per slab: 144, slice oversampling: 6.7, field of view (FOV): 180 mm, bandwidth: 465 Hz/Px, matrix (pixels): 384 × 384, voxel size: 0.5 × 0.5 × 0.8 mm. The areas examined by 3D-CISS were from the basal cistern to the vertex. The midbrain was not included in order to minimise examination time and avoid movement artifacts.

Magnetic resonance angiography (MRA) was performed using a three-dimensional top-of-flight method with multiple overlapping thin slabs acquisition (MOTSA) to increase the resolution for small arteries. Image acquisition time was 8 min 19 s and the parameters were as follows: 9 slabs, slice thickness: 0.5 mm, slice per slab: 30 slices, oversampling: 6.7, TR: 22.0 ms, TE: 3.69 ms, flip angle: 18 degrees, FOV: 200 mm, bandwidth: 199 Hz/Px, matrix (pixels): 323 × 448, voxel size: 0.4 × 0.4 × 0.5 mm. MOTSA was applied to the first 10 studies (5 controls and 5 patients). Due to the time required for data acquisition, MOTSA of three slabs was applied in remaining controls and patients, reducing data acquisition time to 4 min and 46 s.

Fluid attenuated inversion recovery (FLAIR) images were used to evaluate ventricular size, periventricular deep white matter hyperintensities, and lacunar infarcts. Their parameters were as follows: TR: 10,000.0 ms, TE: 90.0 ms, flip angle: 150 degrees, slice thickness: 5 mm, FOV: 230 mm, voxel size: 0.6 × 0.6 × 5 mm, image acquisition time: 2 min 42 s.

Diffusion tensor imaging (DTI) sequence was applied to one healthy individual. Data were acquired with multi-directional diffusion weighting mode in each of 30 different directions with a b-value (diffusion weighting factor) of 1,000 s/mm^2^ and one reference image (b-value of 0). The parameters were as follows: TR: 8,000 ms, TE: 93.0 ms, slice thickness: 2.5 mm, slice per slab: 50, FOV: 240 mm, bandwidth: 1,336 Hz/Px, matrix (pixels): 96 × 96, voxel size: 2.5 × 2.5 × 2.5 mm.

### Image analysis

Ventriculomegaly was defined by an Evans index (maximum width of anterior horn/maximum width of cranium in axial slice) above 0.3. Periventricular and deep white matter hyperintensities were classified based on Fazekas classification [20]. VRS were identified in the basal ganglia and white matter by their tubular, ovoid, or round shape on CISS. VRS were differentiated from lacunar infarcts by a sharply demarcated symmetry of the lesions which lacked a hyperintense rim on FLAIR [21].

Virchow–Robin spaces were classified into three groups according to their numbers; few, moderate and abundant. The classification was defined as follows (per cerebral hemisphere): <10 for the “few” group, 11–30 for the “moderate” group, and >31 for the “abundant” group. The large number of VRS seen in the aged subjects on CISS images necessitated this semiquantitative assessment. The assessment was done in axial sections of the cerebral hemisphere; VRS in the white matter at a level of the semioval center and VRS in basal ganglia at the level of anterior commissure, respectively. Two examiners (Authors: MI and SY) assessed each subject separately and an inter-rater agreement was evaluated statistically.

### Statistical analysis

For the analysis of means for continuous data, the Wilcoxon signed-rank test was used for nonparametric statistics. The percentage comparison of the categorical data was performed using the Chi square test. The threshold for statistical significance was set to 0.05. The inter-rater agreement was examined with Cohens’ kappa coefficient. The values from 0.61 through 0.8 indicated good agreement, and the values >0.81 indicated excellent agreement [22]. All statistical analyses were performed with JMP, version 11 (SAS Institute Inc.).

## Results

Clinical and MRI features of both groups are summarized in Tables 1 and 2. VRS were clearly seen on 3D-CISS images in all individuals in both groups.

**Table 1:** Clinical background of study population

	Control	iNPH	*P* value
Number	9	20	
Male preponderance, N (%)	6 (66.7)	12 (60)	0.7308
Age	74.2 ± 7.9	76.1 ± 6.4	0.6869
History			
Hypertension	5	11	0.9778
Diabetes mellitus	0	2	0.2128
Hyperlipidemia	3	6	0.858
Anti-coagulant/platelet drugs	1	8	0.0988
Lacunar stroke	1	2	0.928
Alzheimer’s disease	0	3	0.1417
Chronic kidney disease	0	1	0.0713
Chronic obstructive pulmonary disease	0	1	0.0713
Activity of daily life			
Modified Rankin scale (G2, 3, 4)/(G0–4) (%)	0	95	<0.0001*
Symptoms (iNPHGS)			
Gait disturbance (G2–4)/(G0–4) (%)	0	85	<0.0001*
Cognition disturbance (G2–4)/(G0–4) (%)	0	85	<0.0001*
Urination disturbance (G2–4)/(G0–4) (%)	0	75	0.0009*
Tap test			
TUG (baseline) (s)	NA	35.0 ± 47.7	
TUG (% change)	NA	25.6 ± 34.6	
MMSE (baseline score)	NA	21.4 ± 5.9	
MMSE (difference)	NA	3.1 ± 2.9	
Shunt surgery, N	0	12	

*G* grade, *iNPHGS* iNPH grading scale, *MMSE* minimental state examination, *NA* not assessed, *TUG* timed up and go test.

* Statistically significant.

**Table 2:** Summary of MRI findings

	Control	iNPH	*P* value
Number	9	20	
Evans index	0.27 ± 0.02	0.34 ± 0.03	<0.0001*
Evans index >0.3, N (%)	9 (0)	20 (100)	<0.0001*
Callosal angle (degree), mean ± SD	106 ± 10.2	66.6 ± 18.1	<0.0001*
Callosal angle <90°, N (%)	0 (0)	18 (90)	<0.0001*
High convexity tightness, N (%)	1 (11)	16 (80)	0.0021*
Enlarged Sylvian fissure, N (%)	0 (0)	16 (80)	0.0003*
Disproportionately enlarged subarachnoid-space hydrocephalus, N (%)	0 (0)	14 (70)	<0.0001*
Deep white matter hyperintensity (Fazekas rating scale)			0.0038*
Grade 0, N	5	1	
Grade 1, N	2	2	
Grade 2, N	0	11	
Grade 3, N	2	6	
Periventricular hyperintensity (Fazekas rating scale)			0.0013*
Grade 0, N	5	0	
Grade 1, N	1	3	
Grade 2, N	3	7	
Grade 3, N	0	10	
Virchow–Robin spaces in semioval center			0.0047*
Grade 1 (few), N	0	10	
Grade 2 (sparse), N	1	5	
Grade 3 (abundant), N	8	5	
Virchow–Robin spaces in basal ganglia			0.1659
Grade 1 (few), N	2	1	
Grade 2 (sparse), N	1	8	
Grade 3 (abundant), N	6	11	

*N* number of individuals.

* Statistically significant.

A Cohens’ kappa value for the inter-rater agreement was 0.699 (*p* < 0.0001) for VRS in the basal ganglia and 0.818 (*p* < 0.0001) for VRS in the white matter, which showed good and excellent inter-rater agreements, respectively [22].

### VRS in the basal ganglia in the control group

**Figure 1:**
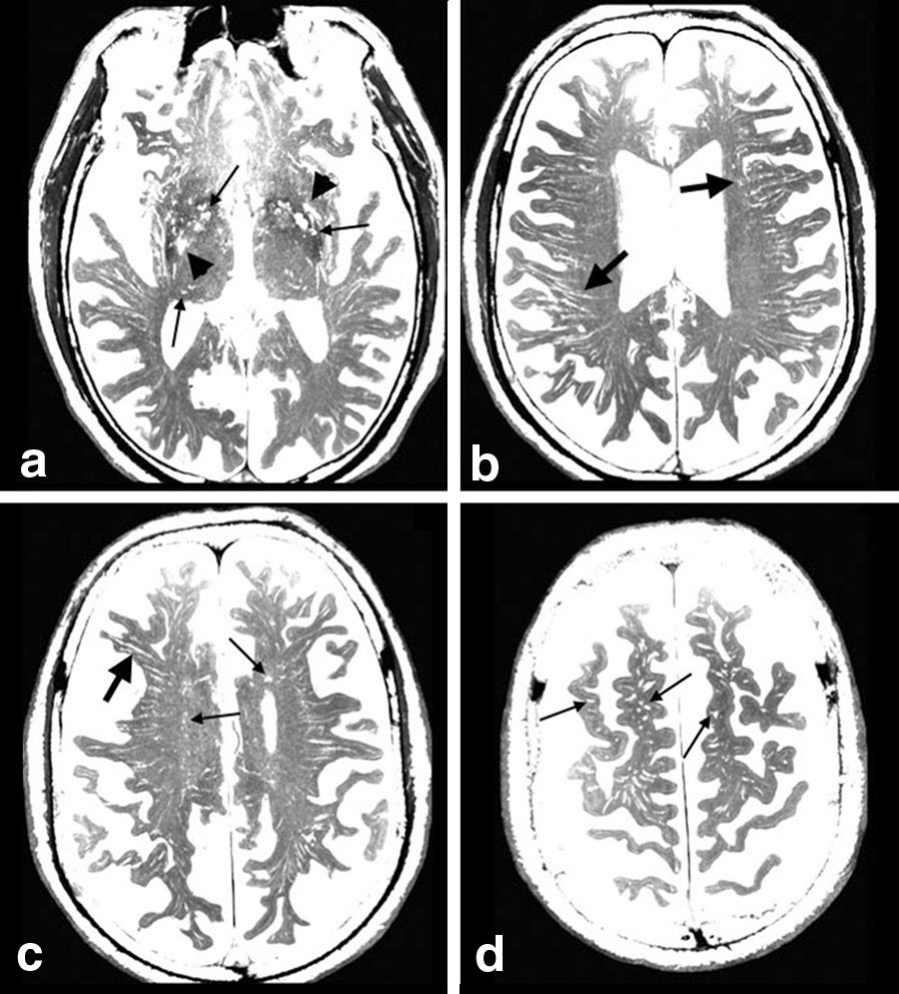
Virchow–Robin spaces (VRS) in axial images in the brain of a control subject. Multi-planar reconstruction constructive interference in steady state (CISS) images: VRS in the basal ganglia (**a**) were round and irregularly sized (*small arrows*). Enlarged VRS sometimes could be seen as oval pools (*arrowheads*) in the lower part of the basal ganglia. In the white matter of the periventricular region (**b**), semioval center (**c**) and subcortical region (**d**), VRS were round (*small arrows*) or tubular (*large arrows*), and homogeneously sized; observation in an 80-year-old woman.

**Figure 2:**
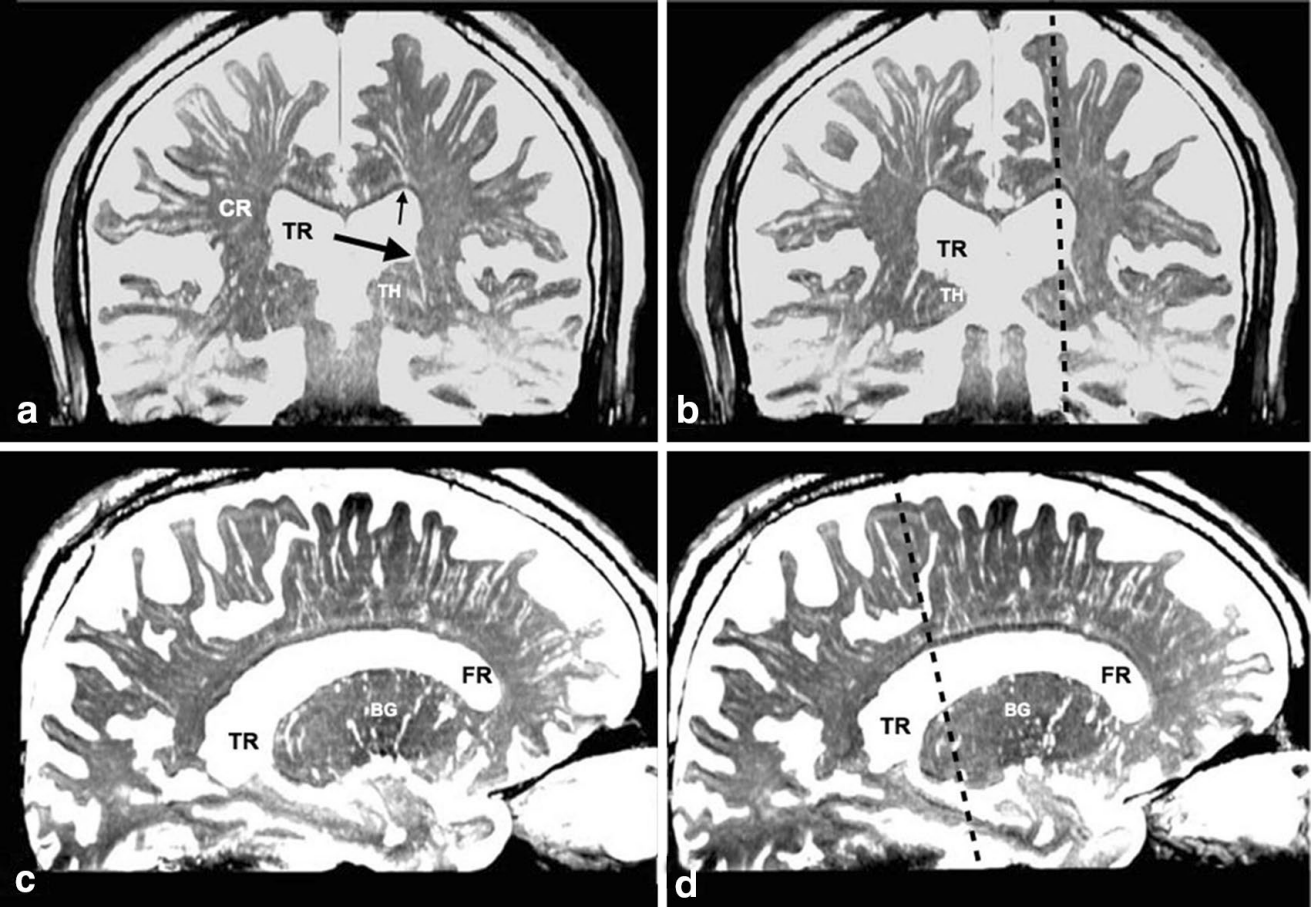
Virchow–Robin spaces (VRS) in coronal and sagittal images in brain of a control subject. **a**, **b** Coronal plane multi-planar reconstruction constructive interference in the steady state (CISS) images. **c**, **d** Sagittal plane multi-planar reconstruction CISS images. VRS in the white matter were divided into medial and lateral groups on coronal sections (**a**, **b**). The medial VRS extended down to the superolateral angle of the lateral ventricle (*small arrow*), while the lateral VRS ran medially and stopped at the lateral end of the corona radiata (CR). In the basal ganglia, VRS extended upward and some of them (*large arrow*) appeared to communicate with the floor of the ventricle. Fluid spaces were also noted just above the corpus callosum. *BG* basal ganglia, *CR* corona radiate, *FR* frontal horn of the lateral ventricle, *TH* thalamus, *TR* trigone of the lateral ventricle, *Dotted* *line* section at the sagittal or coronal image; observation in an 80-year-old woman.

**Figure 3:**
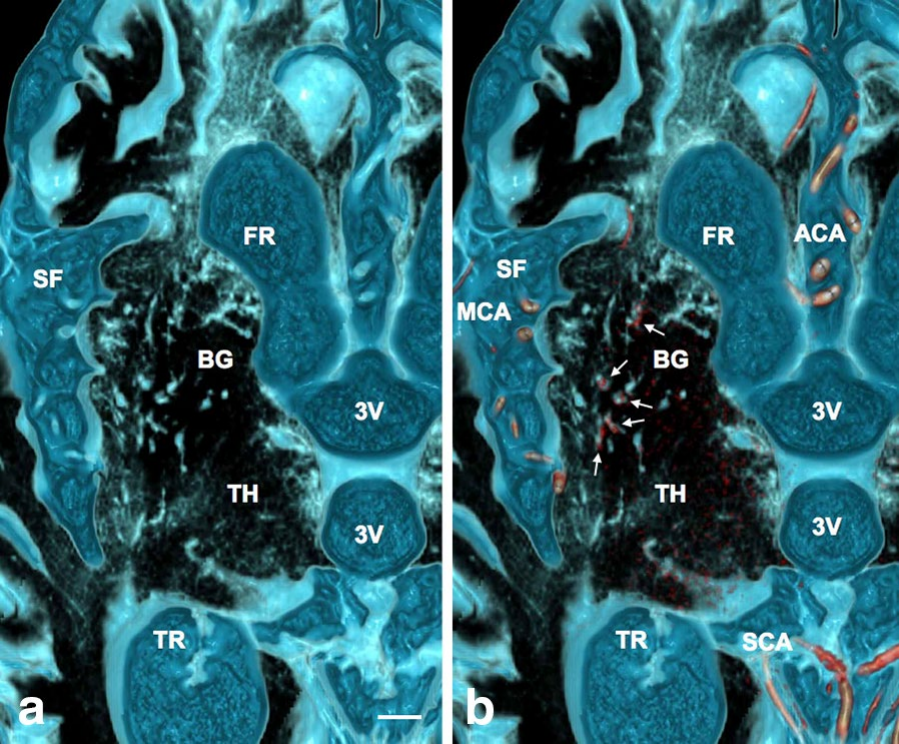
Perforating arteries within Virchow–Robin Spaces (VRS) in the basal ganglia in a control subject. **a** Axial plane volume rendering constructive interference in the steady state (CISS) image. **b** Fusion image of axial plane volume rendering CISS and magnetic resonance arteriography (MRA). In the basal ganglia (BG), VRS were seen as round or oval structures with irregular walls (*light blue*) on CISS image (**a**). On fusion image (**b**), perforating arteries (*red*) were noted in some VRS (*white small arrows*); observations in a normal 62-year-old man. *3V* third ventricle, *ACA* anterior cerebral artery, *SCA* superior cerebellar artery, *FR* frontal horn of the lateral ventricle, *MCA* middle cerebral artery, *SF* Sylvian fissure, *TH* thalamus, *TR* trigone of the lateral ventricle, *scale bar* 5 mm.

**Figure 4:**
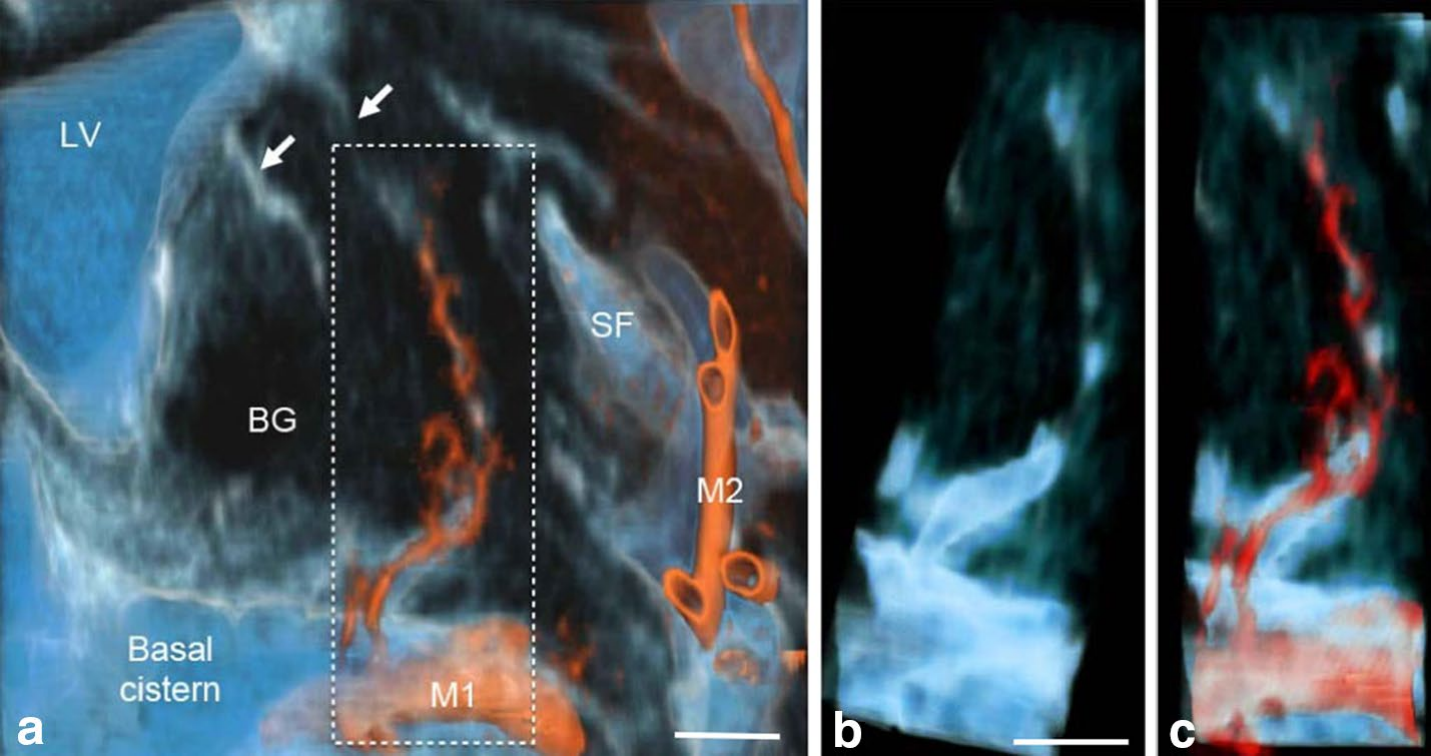
Communication of subarachnoid spaces with Virchow–Robin spaces (VRS) in the basal ganglia of a control subject. **a** Fusion image of coronal plane volume rendering constructive interference in the steady state (CISS) image and magnetic resonance angiography (MRA) image of the basal ganglia. Perivascular CSF spaces were observed around perforating arteries from the M1 portion of the middle cerebral artery (*red*) in the basal ganglia. They passed upward and then medially to the floor of the lateral ventricle (*white arrows*). Magnified view of the *dotted square* (**b**, **c**) showed clear continuity of CSF between the subarachnoid space in the basal cistern and VRS in the basal ganglia on CISS (**b**) and fusion images (**c**). Observation in a normal 69-year-old man, *BG* basal ganglia; *M1*, *M2* M1 and M2 portions of the middle cerebral artery; *LV* lateral ventricle; *SF* Sylvian fissure, *scale bar* 5 mm.

Virchow–Robin spaces seen in the axial plane were round or tubular and irregularly sized in the basal ganglia (Figure 1a–d). Their diameter ranged from 2 mm to more than 5 mm and their number ranged from <10 to >30 in the basal ganglia. In the coronal plane, VRS were semi-curved tubular structures running upward initially and then curved medially to the floor of the lateral ventricle (Figure 2a, b). Their courses were similar to those of the perforating arteries. Their calibers were often irregular (Figure 2a–d). Enlarged VRS could sometimes be seen as oval pools in the lower part of the basal ganglia. CISS and MRA fusion images showed that VRS communicated with subarachnoid CSF and perforating arteries were seen within some of them (Figures 3, 4). Arteries were most commonly seen in VRS in the lower half of the basal ganglia.

### VRS in the white matter in control group

**Figure 5:**
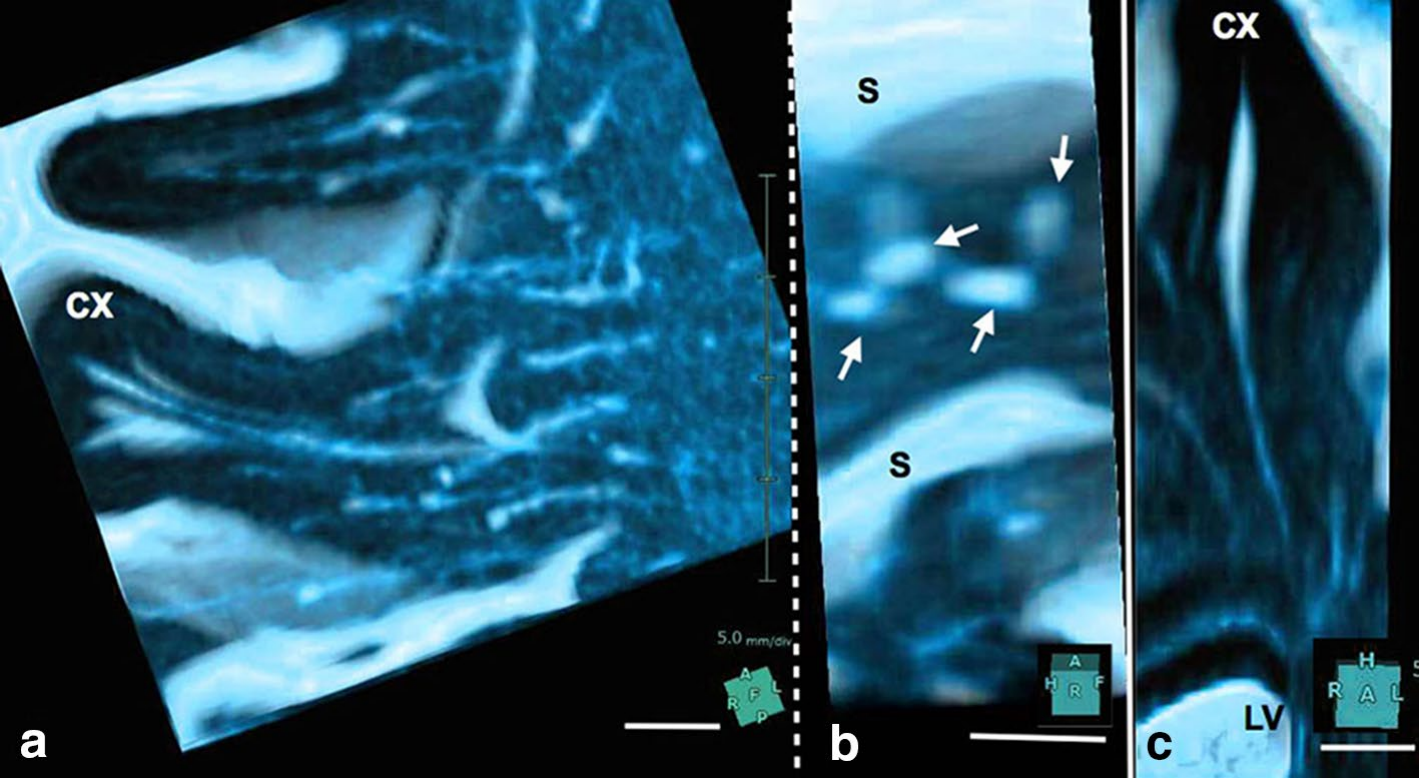
Magnified view of Virchow–Robin spaces in a single gyrus in control subject. **a**, **b** Volume rendering image of a right middle frontal gyrus. **c** Volume rendering image of a left superior frontal gyrus. Several tubular structures with sharp tips and smooth walls were observed in a single gyrus (**a**). They originated at the corticomedullary junction, ran medially, and terminated at the lateral end of the corona radiata. They did not penetrate the cortex (CX). Their sharp tips did not fuse and some disappeared shortly in the subcortical white matter. A view rotated 90° (**b**) from image (**a**) showed several tubular structures (*small arrows*) through the increased transparency of the cortex and subarachnoid cerebrospinal fluid (CSF). In a coronal image (**c**) of the superior frontal gyrus, VRS originated at the corticomedullary junction and ran down to the superolateral angle of the lateral ventricle (LV). Their wall was smooth and mildly curved with some bulging. They did not penetrate the cortex (CX). Observation in a normal 69-year-old man, *S* cerebral sulcus, *scale bar* 5 mm.

**Figure 6:**
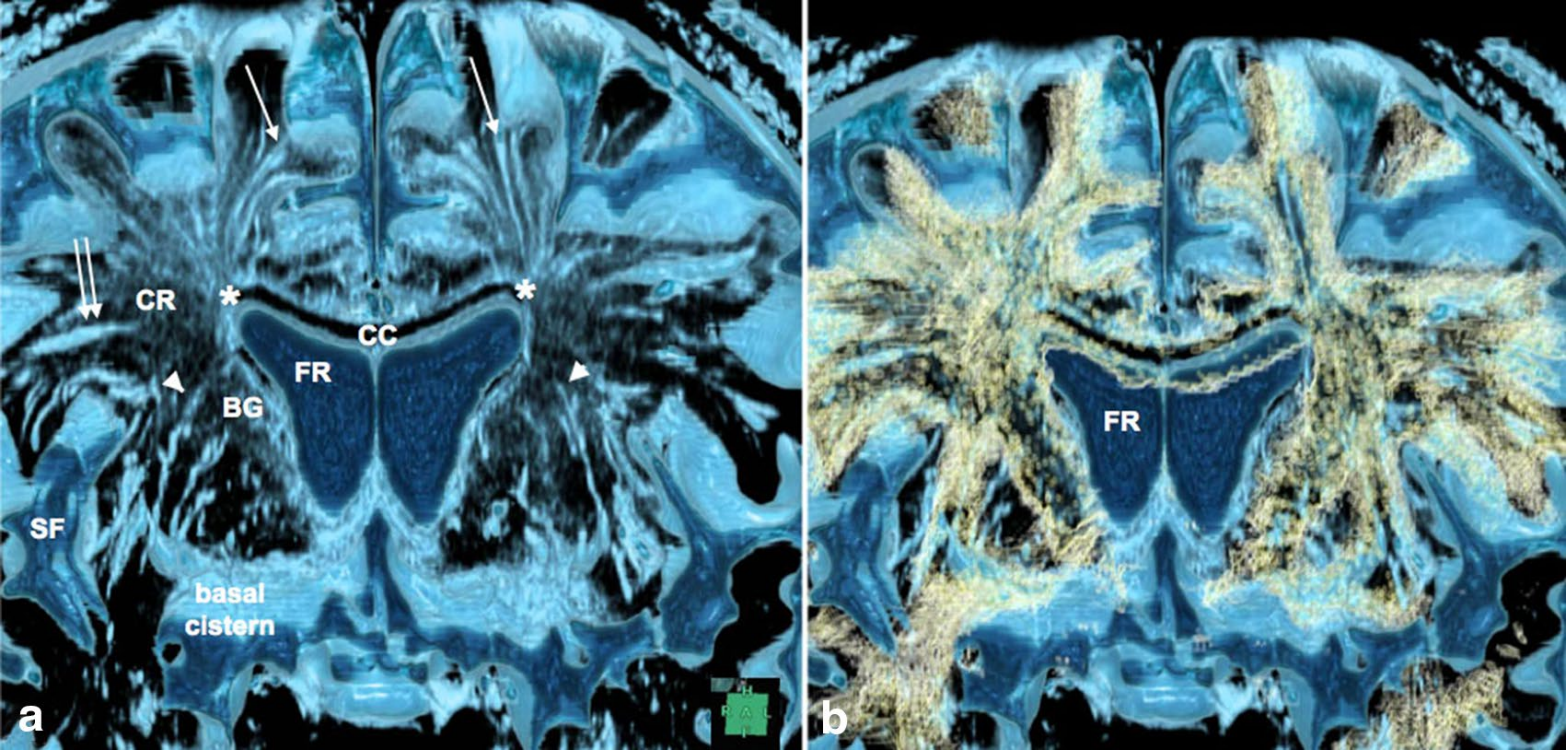
Virchow–Robin spaces (VRS) in the cerebral hemisphere and association with fiber tracts in a control subject. **a** Coronal plane volume rendering constructive interference in the steady state (CISS). **b** Fusion images of CISS and diffusion tensor imaging (DTI) of the cerebral hemisphere. CISS image (**a**) showed medial VRS (*single arrows*) in the white matter extending down to the superolateral angle (*asterisk*) of the lateral ventricle, while lateral ones (*double arrow*) ended at the lateral end of the corona radiata (CR). VRS in the basal ganglia (*arrowheads*) ran upward and curved medially to the floor of the lateral ventricle. Fusion image (**b**) showed VRS in the white matter running parallel to axon tracts (*yellow*), while VRS in the basal ganglia crossed fiber tracts of the internal capsule. Observation in 69-year-old man, *BG* basal ganglia, *CC* corpus callosum, *FR* frontal horn of the lateral ventricle, *SF* Sylvian fissure.

**Figure 7:**
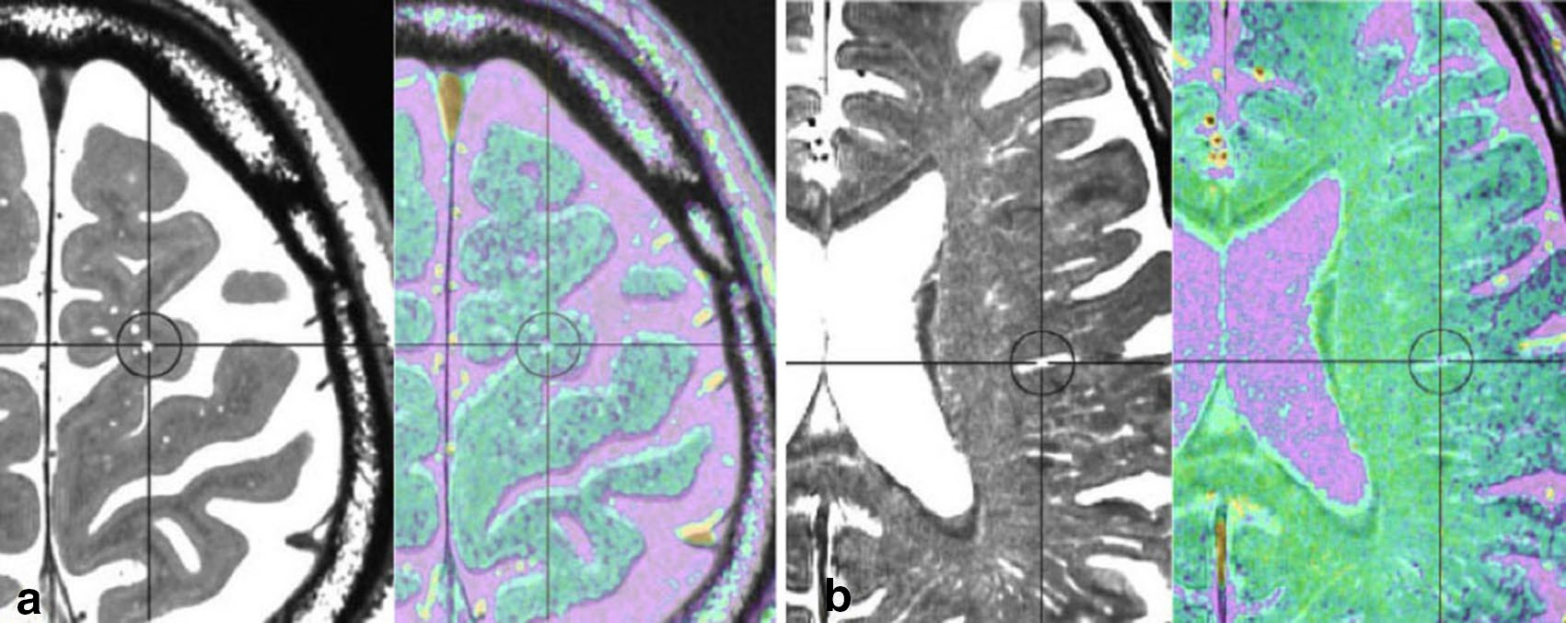
Absence of an artery in the Virchow–Robin spaces (VRS) in the white matter in a control subject. Multi-planar reconstruction constructive interference in the steady state (CISS) image (*black and white*) and magnetic resonance angiography (MRA) fusion image (*color*) in the white matter. CISS image showed VRS as round or tubular structures (*circles*) at subcortical (**a**) and periventricular (**b**) levels. Fusion image did not show any arteries in VRS; observation in a normal 69-year-old man.

In the axial plane, VRS seen in the white matter were homogeneously sized and tubular at the level of the lateral ventricle (Figure 1b, c), and round or oval at the higher level (Figure 1d). VRS were evenly distributed in the white matter but tended to be more numerous posteriorly. Their diameter was ~3 mm with a frequency of >30 per hemisphere. In the coronal and sagittal planes, VRS were tubular with smooth walls (Figure 2). They began at the corticomedullary junctions as pencil-like tips with medial VRS running inferiorly and lateral VRS running horizontally (Figure 2a, b). They did neither penetrate the cortex nor communicate with subarachnoid CSF in 3D images (Figure 5). The length of VRS was variable. Magnified view showed that most of them were short and located mainly near the corticomedullary junction (Figure 5a, b). Some of them were long enough to reach the superolateral angle of the lateral ventricle (Figures 5c, 6). The diameter of VRS was homogeneous in general, but the magnified view showed more dilatation near the corticomedullary junction (Figure 5c). CISS and MRA fusion images showed no perforating arteries within VRS (Figure 7). CISS images showed that the medial ones directed to the superolateral angle of the lateral ventricle and the lateral ones ended at the points lateral to the internal capsule (Figure 6a). CISS and DTI fusion images (Figure 6b) showed VRS in the white matter running parallel to axon tracts, while VRS in the basal ganglia crossed fiber tracts of the internal capsule.

### VRS in iNPH

In the iNPH group, daily activity was significantly impaired and symptom severity was high (Table 1). The tap test showed an average of ~27% gait improvement or ~3-point increase on the MMSE. On MRI (Table 2), ventriculomegaly (Evans index >0.3), high convexity tightness and enlarged Sylvian fissure were noted in 100, 80 and 80% of patients, respectively. DESH was noted in 70% of patients. Higher degrees (Grade 2, 3) of periventricular and deep white matter hyperintensities were more frequent in the iNPH group.

**Figure 8:**
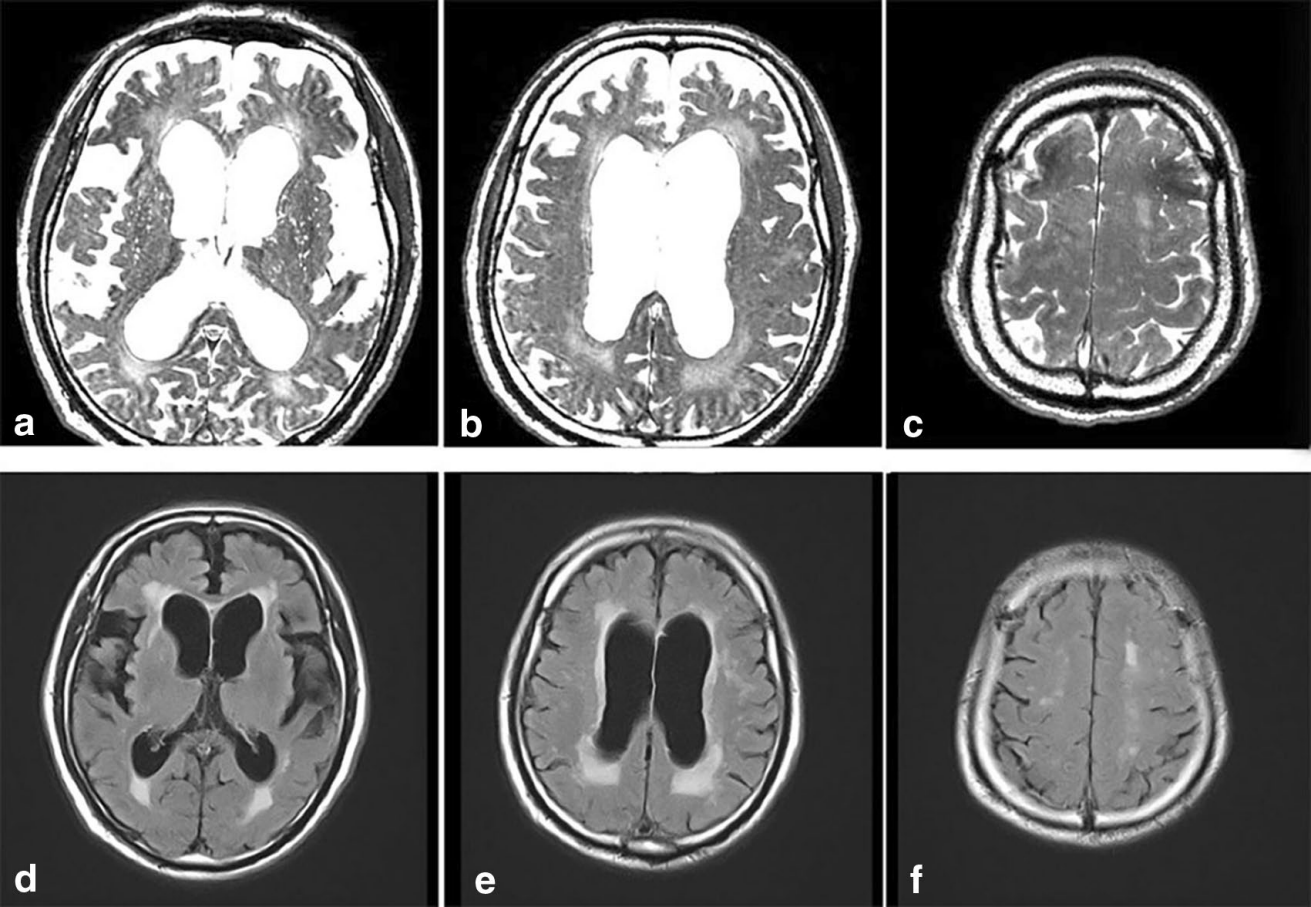
Virchow–Robin spaces (VRS) in an 82-year-old patient with idiopathic normal pressure hydrocephalus (iNPH). In iNPH, constructive interference in the steady state images (**a**–**c**) showed few VRS in the white matter, in contrast to abundant VRS in the basal ganglia. A moderate degree of hyperintensities was noted in the periventricular and semioval center white matter. Corresponding images of fluid attenuated inversion recovery (FLAIR) are shown in **d**–**f.**

VRS were noted in all patients, but tended to be few or moderate in number (Figure 8). For the white matter, there was a statistically significant larger number of patients with few or sparse VRS (*p* < 0.01, Grade 1, 2), but this was not the case in the basal ganglia (Table 2) where there were no marked differences in diameter and length between control and iNPH.

**Figure 9:**
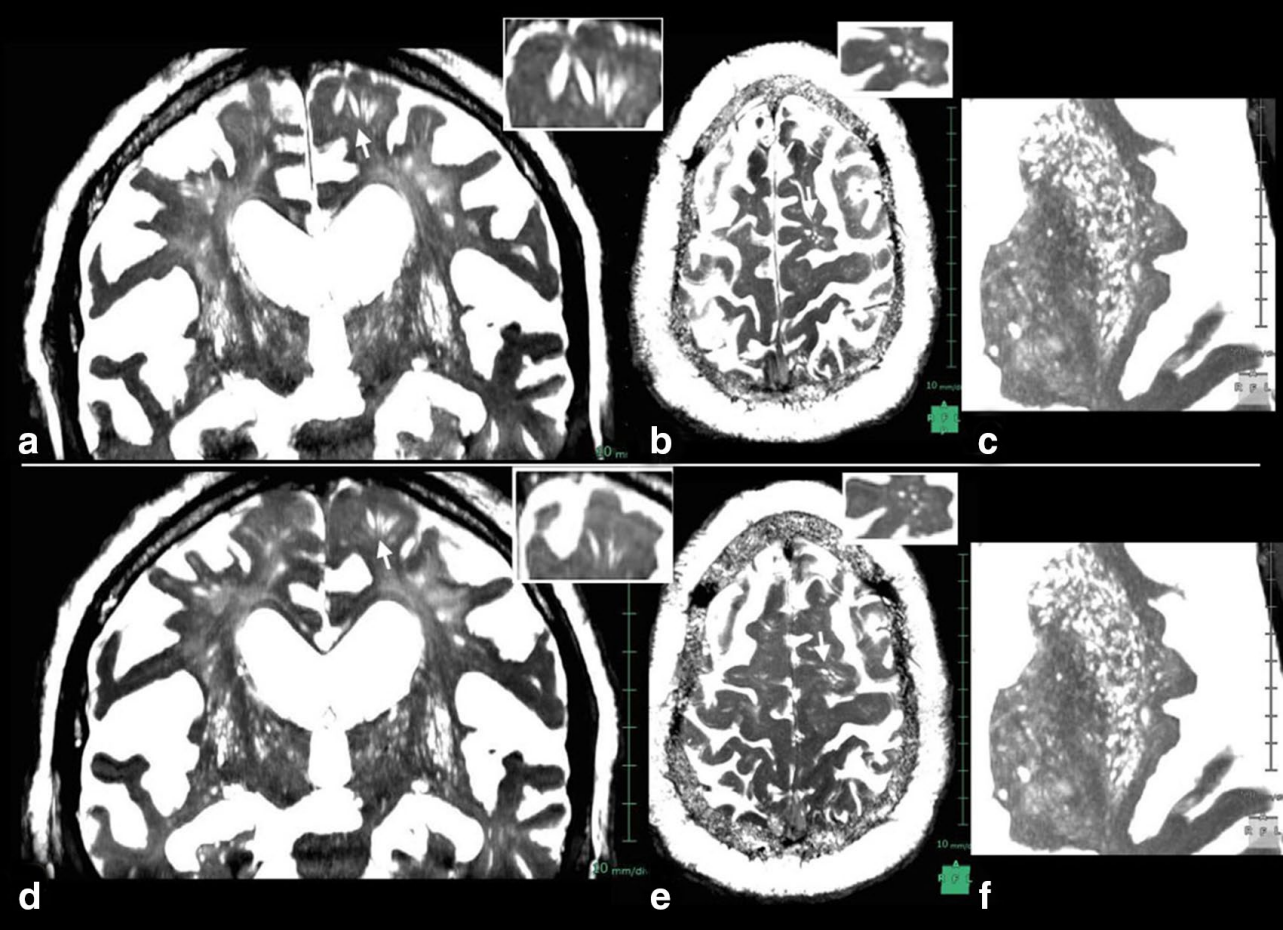
Postoperative constructive interference in the steady state images 4 months after shunt surgery in a patient with iNPH. Preoperative images (**a**–**c**) and postoperative images (**d**–**f**); **a**, **d** coronal sections, **b**, **e** axial sections at the level of corticomedullary junction. **c**, **f** axial sections at the level of basal ganglia. *Inserts* at *right upper corner* are magnified views of *arrow*-indicated VRS. Short-stepped gait was improved immediately after surgery. The CISS images 4 months after surgery showed mild decrease in VRS diameter in the white matter, although there were no significant changes in number. No significant changes were noted in basal ganglia VRS, observation in a 68-year-old patient with iNPH.

Postoperatively, the symptoms, especially of gait disturbance, were improved after shunt surgery in all patients treated. Follow-up MRIs were taken 1–10 months after surgery in four patients. On magnified view of the CISS images, a small decrease in VRS diameter was noted in the white matter, especially near the corticomedullary junction, although there were no significant changes in VRS number (Figure 9). In the basal ganglia, there were no significant changes in VRS number and diameter.

## Discussion

Virchow–Robin spaces are fluid-filled spaces surrounding perforating arteries in the brain parenchyma. Enlarged VRS have long been regarded as benign normal variants, but can also be seen in various pathological disorders [3, 6–9]. In this study, 3D-CISS sequences on 3-T MRI, which are very sensitive to brain water content, enabled the observation of fluid-filled spaces and three-dimensional observation clearly revealed the fine morphological features of VRS. We noted marked differences between VRS in the basal ganglia and white matter. First, VRS in the basal ganglia were irregularly sized, with some parts dilated and others markedly narrowed. Second, arteries were seen within VRS, especially in the ventral of the basal ganglia. Third, VRS communicated with CSF in the basal cistern. Fourth, VRS crossed the fiber tracts of the internal capsule. Fifth, their course in the basal ganglia corresponded well to perforating arterial trajectories.

Virchow–Robin spaces in the white matter differed from those in the basal ganglia in several ways. First, VRS in the white matter were noted to be in the whole hemispheric areas, and were homogeneously sized with smooth walls. Second, arteries were not seen inside VRS of the white matter. Third, these VRS did not communicate with CSF in the subarachnoid spaces. Fourth, they ran parallel to fiber tracts. The medial VRS ran towards the superolateral angle of the lateral ventricle, while lateral VRS ran medially and stopped at the lateral part of the internal capsule. Fifth, VRS originated at the corticomedullary junction and it must be noted that VRS did not penetrate the cortex. Thus, morphological feature differences between VRS of the basal ganglia and white matter were remarkable.

Using the same CISS sequence with 3T-MRI, Tsutsumi et al. [4] studied VRS in 105 control subjects and showed that the cortical segment of VRS were observed as the fine pores in the cerebral cortex, communicating with the subarachnoid cisterns, and coursing subcortically. However, VRS in their axial images would be in the corticomedullary junction, not in the cortex. On their coronal and sagittal images, VRS did not show any communication with the subarachnoid space. In the present study, 3-D CISS sequences on 3T-MRI did not show any VRS in the cortex, and high resolution MRA did not show small arteries within VRS in the white matter. However, perforating arteries within VRS in the basal ganglia were clearly observed in the ventral half of the basal ganglia. These different results may be due to the limitations of MRI resolution. Bouvy et al. [23], using a 7T-MRI, reported smoothly shaped perivascular spaces in the semioval center, corresponding to VRS. These originated a few millimeters below the cortex, converged and tapered toward the ventricle, and ended 1–2 cm before the ventricle wall. They did not observe any extensions of VRS into the cortex. Their description corresponds well to our observations. They also noted that VRS correlated spatially with lenticulostriate and perforating arteries in the semioval center, but not with veins. Actually, the pattern of VRS in the white matter and basal ganglia is similar to that of medullary arteries in the brain, but not of veins [24], although VRS in the white matter did not penetrate the cerebral cortex.

There are several reports on differences between VRS of the white matter and basal ganglia. Pollock et al. [25] reported a difference in perivascular structures between the basal ganglia and cerebral cortex under ultramicroscopic observation. Charidimou et al. [8] compared the frequency of VRS in patients with or without cerebral amyloid angiopathy, and found more VRS in the white matter of patients with cerebral amyloid angiopathy. In contrast, we found no difference in VRS of the basal ganglia between the control and iNPH patients groups. Adachi et al. [26] reported that VRS were confined to a fixed level in the lower part of the basal ganglia, and not found near the brain surface. Recently, Weller et al. [27] proposed a mechanism of cerebral amyloid angiopathy–induced dilatation of perivascular spaces in the white matter. In their review, they presented an illustration of a penetrating artery where the VRS did not dilate in the cortex but dilated in the white matter due to retention of the interstitial fluid. No penetration of VRS in the cortex in our observation would correspond with their illustration. Roher et al. [28] suggested that dilation of VRS in the white matter in Alzheimer’s disease may be associated with the deposition of Aβ in the perivascular fluid drainage pathways of cortical and leptomeningeal arteries. Thus, we may observe cerebral amyloid angiopathy on CISS images, even in aged subjects maintaining normal daily activities. Barkhof [29] suggested that enlarged VRS in the white matter might represent local atrophy, independent of cortical atrophy and ventricular widening. Inglese et al. [8] reported higher numbers of VRS in mild traumatic brain injury, compared to control, that increased with age. Their findings were suggestive of mild local axon atrophy. Abbott [30] proposed the possibility of ISF-CSF mixture through spaces between axon tracts. Taken together, VRS seen in the white matter on MRI or CT may be spaces between axon tracts containing ISF-CSF mixtures. This might be a reasonable explanation for an increase of enlarged VRS in aged populations and various disorders associated with local axon tract atrophy. In this case, VRS in the white matter may be renamed dilated ISF spaces. As stated above, VRS in the white matter tend to increase in number with normal aging and various brain disorders. In contrast, Akiguchi et al. [31] reported that VRS in the white matter were significantly decreased in patients with suspected iNPH compared to patients with various other pathologies, such as brain atrophy, lacunar infarcts, Binswanger disease, Alzheimer’s disease, and Parkinson’s disease. The present results are consistent with their findings regarding white matter VRS. In contrast to the white matter VRS, there were no significant changes in the basal ganglia VRS between control and iNPH groups. Also, no significant changes were noted between pre- and post-operative patients. These findings may indicate that basal ganglia would not be responsible for gait disturbance such as short-stepped gait or frozen gait.

There are several possibilities for the sparseness of white matter VRS in patients with iNPH. The first one is dilated ISF spaces of local axon tract atrophy with ISF drainage pathway. They could be compressed by higher tissue pressure in the white matter in iNPH. This possibility is supported by our observations in the postoperative follow-up, where the VRS decreased in size in the white matter. The second possibility is cerebral amyloid angiopathy-induced dilatation of VRS. In iNPH, ventricular dilatation would be closely related to increased pulsation of ventricles which compress the white matter more than the cortex. This could prevent dilatation of white matter VRS even with obstruction of ISF drainage pathway in the cortical arterioles and leptomeningeal arteries. Even if compression is relieved after surgery, amyloid deposition in white matter VRS would not develop so rapidly. At present, it remains unclear whether the white matter VRS is dilated ISF spaces or cerebral amyloid angiopathy, or both. Further studies are necessary to clarify the functional role of VRS in normal subjects and patients with iNPH.

## Conclusion

The fine morphological features of VRS in the basal ganglia and white matter were studied with 3-D CISS sequences on 3-T MRI in normal aged subjects and patients with suspected iNPH. VRS in the basal ganglia are genuine perivascular spaces, whereas VRS in the white matter may represent spaces between axon tracts. In most patients with iNPH, there were few or no VRS in the white matter. In contrast, no significant changes were noted in basal ganglia VRS. At present, it remains unclear whether the white matter VRS is dilated ISF spaces or cerebral amyloid angiopathy, or both. Further studies are necessary to elucidate the pathophysiological role of VRS in normal subjects and in various disorders, including iNPH.

## Abbreviations

3D: three-dimensional
3-T: 3 Tesla
3V: third ventricle
BG: basal ganglia
CISS: three-dimensional constructive interference in the steady state
CR: corona radiata
CX: cortex
CSF: cerebrospinal fluid
DESH: disproportionately enlarged subarachnoid-space hydrocephalus
DTI: diffuse tensor imaging
FLAIR: fluid attenuated inversion recovery
FOV: field of view
iNPH: idiopathic normal pressure hydrocephalus
iNPHGS: idiopathic normal pressure hydrocephalus grading scale
ISF: interstitial fluid
LV: lateral ventricle
M1: M1 portion of middle cerebral artery
M2: M2 portion of middle cerebral artery
MOTSA: multiple overlapping thin slabs acquisition
MRI: magnetic resonance imaging
MRA: magnetic resonance angiography
mRS: modified Rankin scale
SA: subarachnoid space
SF: Sylvian fissure
TE: echo time
TR: repetition time
VRS: Virchow–Robin spaces
